# A retrospective natural history study in adult and juvenile patients with incident dermatomyositis and polymyositis using real world data

**DOI:** 10.1007/s10067-025-07614-6

**Published:** 2025-08-28

**Authors:** David M. Barnes, Daniela Graham, Cecilia E. Borlenghi, Thomas Edwards, Stephen E. Schachterle, Helen Sile

**Affiliations:** 1 Pfizer Inc, MS 66HB-13N251/261, 66 Hudson Boulevard East, New York, NY 10001 USA; 2 Pfizer Inc, Groton, CT USA; 3 Pfizer Inc, Buenos Aires, Argentina; 4 Panalgo Inc, Boston, MA USA

**Keywords:** Dermatomyositis (DM), Incidence rates, Juvenile dermatomyositis (JDM), Juvenile polymyositis (JPM), Natural history, Polymyositis (PM)

## Abstract

**Objective:**

This retrospective natural history study used real-world data to describe baseline demographics, comorbidities, clinical characteristics, and treatments, and assess incidence rates (IRs) of extra-muscular outcomes in patients with incident dermatomyositis (DM), polymyositis (PM), juvenile DM (JDM), and juvenile PM (JPM).

**Methods:**

De-identified clinical data were collected from the US Optum® electronic health records with supplemental claims (01 January 2016 to 31 March 2021). Total 9,009 patients were included (DM: 4,275; PM: 4,559; JDM: 128; JPM: 47). IRs of 13 outcomes were estimated in patients and an equal number of sex- and age-matched controls (MCs) without DM/PM.

**Results:**

Mean age at index was 54.5 (DM), 57.3 (PM), 14.3 (JDM), and 15.1 years (JPM). Most common comorbidities were hypertension in DM (50.3%) and PM (63.9%) cohorts, dysphagia in JDM (15.6%) and liver disease in JPM (23.4%) cohorts. Most common clinical characteristics were Raynaud’s phenomenon in DM (8.6%), PM (7.9%), and JDM (11.7%) cohorts, and arthritis in JPM (10.6%) cohort. Systemic steroids were the most frequent medication (DM: 70.3%; PM: 68.3%; JDM: 73.4%; JPM: 59.6%). IRs (per 100 person years) of outcomes in all cohorts were higher in patients versus their MCs. In DM and PM cohorts, highest IRs were observed for gastroesophageal reflux disease (DM:10.3; PM:12.8). In JDM cohort, dysphagia (4.3) had highest IR. In JPM cohort, cardiac dysrhythmia (3.5) had highest IR.

**Conclusion:**

This study addresses existing gaps in understanding the descriptive epidemiology of DM and PM in the US, particularly the IRs of extra-muscular disease manifestations and malignancy events.

**Supplementary Information:**

The online version contains supplementary material available at 10.1007/s10067-025-07614-6.

**Table:** 

**Key Points** • *This study provides a comprehensive description of demographic and disease characteristics in a large and nationally representative sample of adult and juvenile patients with incident DM and PM in the US.* • *The results cover wide arrays of demographics, comorbidities, clinical characteristics, treatment exposures, and extra-muscular disease manifestations and malignancies in patients with incident DM and PM.* • *The results frequently align with findings from other, smaller studies in patients with myositis.*	

## Introduction

Dermatomyositis (DM) and polymyositis (PM), two main sub-types of idiopathic inflammatory myopathy (IIM), are rare autoimmune diseases characterized by proximal skeletal muscle weakness and muscle inflammation [1]. Extra-muscular manifestations include affected joints, lungs, gastrointestinal tract, and heart [2]. DM also leads to skin manifestations, such as rash or ulceration [1]. The prevalence and incidence of DM and PM vary across countries, with relatively high estimates of both in the US.

Country-specific annual prevalence estimates of adult DM and PM range from 2.0 (Australia) to 9.2 (US) [3, 4] and 2.1 (South Korea) to 11.2 (US) [3, 5] cases per 100,000 persons, respectively. Country-specific annual incidence estimates of adult DM and PM range from 0.2 (South Korea) to 1.5 (US) [3, 5] and 0.3 (South Korea) to 3.8 (US) [5, 6] cases per 100,000 persons, respectively. DM and PM are more prevalent in females compared with males [3, 4, 6–11] and both conditions are less prevalent in children than in adults [10, 12, 13], with juvenile PM being particularly rare [10].

Treatment options for DM and PM are limited [14]. Frequent use of off-label treatment regimens lacking adequate data from well-controlled clinical trials has safety concerns, and a majority of patients are treatment refractory [15–17]. To date, only one FDA-approved therapy, intravenous immunoglobulin (IVIG), is available to treat adult DM [15, 16, 18].

The rarity of these conditions has hindered the recruitment of large study cohorts in which the diseases can be robustly characterized [8], thereby precluding a more complete understanding of their pathophysiology, epidemiology, patient demographics, clinical characteristics, treatment exposures, and disease progression patterns, including the extent of extra-muscular organ manifestation and risk of malignancies [19, 20]. These gaps in understanding warrant additional studies [9, 14, 16, 17, 21].

The current study addresses the limitations of the previous studies by comprehensively assessing the demographics, comorbidities, clinical characteristics, treatment patterns, and extra-muscular disease manifestation rates in a single large sample, which to our knowledge, is the largest cohort of DM and PM patients assembled to date. Additionally, rates of extra-muscular disease manifestations and malignancies are compared with rates in matched controls to better contextualize disease burden in DM and PM patients. The study was conducted using the US Optum Market Clarity, Electronic Health Records (EHR) with supplemental claims database. This US EHR database has a large patient sample from all 50 states in the US [22], allowing for robust estimates fairly generalizable to disease characteristics and outcomes in the US population. It is important to note that DM and PM are increasingly recognized as heterogeneous disease categories [23]. However, diagnostic coding has not yet fully accounted for the identification of these specific subtypes. Therefore, this study relies on the diagnostic coding practices used during the study period.

The findings of this study may help healthcare providers better anticipate disease progression and tailor treatment plans accordingly. Additionally, this study may generate data to support the clinical development of appropriate targeted therapeutic options.

## Materials and methods

### Data source

Data were collected from the Optum Market Clarity US database, which links EHR data with supplemental medical and pharmacy claims from providers across the continuum of care. The study was compliant with the Health Insurance Portability and Accountability Act (HIPAA), statistician-certified, and contains de-identified data of commercial health plan members and Medicare and Medicaid Advantage members. The dataset includes EHR elements, such as lab results, vital signs and measurements, diagnoses, and procedures [22]. Diagnostic, laboratory, prescription, and surgical procedure data were obtained using International Classification of Diseases, Tenth Revision, Clinical Modification (ICD-10-CM), International Classification of Diseases, Ninth Revision/Tenth Revision, Procedure Classification System (ICD-10-PCS), National Drug Centre (NDC), Healthcare Common Procedure Coding System (HCPCS) codes, or Current Procedural Terminology (CPT) codes, as applicable [24].

Since this study used anonymized structured data that is not subject to privacy laws, informed consent from patients or Institutional Ethics Committee (IEC)/Institutional Review Board (IRB) approval was not required. The study was conducted in accordance with practices described in Guidelines for Good Pharmacoepidemiology Practices (GPP) by the International Society for Pharmacoepidemiology (ISPE), European Medicines Agency (EMA), European Network of Centers for Pharmacoepidemiology and Pharmacovigilance (ENCePP), and Guide on Methodological Standards in Pharmacoepidemiology and other applicable laws and regulations.

### Study design and population

**Fig. 1:**
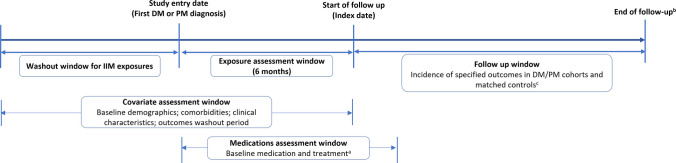
Study design. **a** Baseline medication and treatment were assessed during a 7-month period: the exposure assessment window plus one month. **b** Follow-up ended at either death, end of database activity, a post-index diagnosis of DM or PM other than the cohort-defining diagnosis or any DM or PM diagnosis for matched controls, or end of the study period, whichever occurred first. Follow-up for an incident event ended at its first occurrence. **c** Comparison groups of matched controls (with no diagnosis of DM/PM) was matched to DM or PM patients based on age, sex, and index month/year. DM, dermatomyositis; IIM, idiopathic inflammatory myositis; PM, polymyositis

The study period was 01 January 2016 to 31 March 2021. For inclusion in the study, patients must have been receiving care through an Integrated Delivery Network (IDN) which improves the record capture across differing clinical settings compared to non-IDN settings. Patients must have had continuous activity in the database for ≥ 6 months before and ≥ 6 months after their first IIM diagnosis of the study period. They cannot have had an IIM diagnosis at any point in the database prior to their first IIM diagnosis in the study period. IIM symptoms may not initially present with a clinical profile that is sufficiently clear for a definitive diagnosis, which may only be possible after more symptoms emerge over time. Therefore, a 6-month ‘exposure assessment window’ (EAW), starting with the first inpatient or outpatient DM or PM diagnosis during the study period, was added to allow for an alternative diagnosis to emerge (Fig. 1). During the EAW, an algorithm assigned 2 counts to each inpatient DM and PM diagnosis and 1 count to each outpatient DM and PM diagnosis. At the end of the EAW, patients were assigned to a DM or PM cohort (or neither) based on which subtype had the highest count. If DM and PM counts were equal, patients were assigned to the cohort of their last diagnosis during the EAW. Patients with only outpatient diagnoses must have had ≥ 2 outpatient diagnoses of the same subtype to be assigned to a cohort.

The index date, which initiated the follow up period, was defined as the day after the end of the EAW (Fig. 1). Only patients aged ≥ 12 years were enrolled; those aged 12 to < 18 years at the index date were assigned to the respective JDM or JPM cohort and patients aged 18 years or older at the index date were assigned to the respective adult DM or PM cohort.

Matched controls (MCs) with no diagnosis of adult or juvenile DM or PM were randomly selected and matched one-to-one without replacement with the adult or juvenile DM or PM patients. Each DM and PM patient was matched with one control on birth year and natal sex (female or male), and follow-up began in the same month and year. DM and PM patients and their MCs were followed up until whichever of the following occurred first: death, end of database activity, a post-index diagnosis (1 inpatient or 2 outpatient) of DM or PM other than the cohort-defining diagnosis (or any DM or PM diagnosis for MCs), or end of the study (31 March 2021).

### Baseline characteristics

The baseline characteristics assessed were demographics, comorbidities, clinical characteristics, and prescribed medications and treatments. Demographics were defined using the last observed value during the 12-month period prior to the index date. Comorbidities and clinical characteristics were identified during the 12-month period prior to the index date. Medications and treatments were assessed during a 7-month period (6-month EAW plus the first month post-index date).

### Outcomes of interest

Extra-muscular incident outcomes of interest were selected a priori based on clinical expertise among the study authors, consultation with key opinion leaders, and the review of published literature. These outcomes were identified during the follow-up period and consisted of core extra-muscular manifestations of DM or PM, as well as malignancies, and all-cause death. Due to the association between malignancy and DM and PM in many epidemiologic studies, malignancy was one of the incident events of interest. Follow-up for a specific event ended at its first occurrence during the post-index period. Patients identified with an event during the 12-month period prior to the index date (the washout period) were not followed-up for that event but were followed up for other events.

### Statistical analysis

For all cohorts and MCs, continuous (mean or median) and categorical (counts and percentages) variables were summarized and crude incidence rates (IRs) of outcome events were estimated. Because this is an exploratory rather than hypothesis-testing study, multiple testing corrections were not performed in our estimates of incidence rates. For each outcome event, progression times were measured as the mean time from index to the occurrence of that event and percentage of patients in whom the event occurred were estimated at 6 months, 1 year, and 2 years. Binary classification random forest algorithms were created for each of interstitial lung disease (ILD), malignancies, and all-cause death outcomes in adult DM and PM cohorts. Potential predictors were the demographics, comorbidities, and clinical characteristics measured during the 12-month period prior to the index date (listed in Tables 1 and 2), and the medical and treatment variables measured during the 6-month EAW and subsequent first month post-index date (listed in Table 3). The parameters used for random forest models for ILD, malignancies, and all-cause death in DM and PM cohorts were AUC (area under the curve), 95% CIs around AUC, recall, precision, negative predictive value, and specificity. For each model, data from 70% of randomly selected patients were used for validation and data from the remaining 30% of patients were used for training. The Mean Decrease in Impurity (MDI) metric, which corresponds to the Gini Index, was used to determine the top predictors in the random forest models. All data management was performed with Panalgo Inc. Instant Health Data (IHD)® and additional analyses were conducted using R [25].

**Table 1:** Baseline demographic characteristics by cohort

Demographic characteristics^a^	Cohorts			
	DM (*n* = 4,275)	JDM (*n* = 128)	PM (*n* = 4,559)	JPM (*n* = 47)
Age, mean (SD)	54.5 (15.5)	14.3 (1.7)	57.3 (15.5)	15.1 (1.7)
Sex, n (%)				
Female	3,253 (76.1)	89 (69.5)	2,849 (62.5)	26 (55.3)
Male	1,022 (23.9)	39 (30.5)	1,710 (37.5)	21 (44.7)
Race, n (%)				
Asian	83 (1.9)	2 (1.6)	72 (1.6)	2 (4.3)
Black	429 (10.0)	16 (12.5)	856 (18.8)	11 (23.4)
Caucasian	2,928 (68.5)	62 (48.4)	2,692 (59.1)	17 (36.2)
Unknown	835 (19.5)	48 (37.5)	939 (20.6)	17 (36.2)
Ethnicity, n (%)				
Hispanic	245 (5.7)	14 (10.9)	244 (5.4)	5 (10.6)
Non-Hispanic	2,983 (69.8)	65 (50.8)	3,072 (67.4)	28 (59.6)
Unknown	1,047 (24.5)	49 (38.3)	1,243 (27.3)	14 (29.8)

^a^ Assessed during the 12-month period prior to the index date

*DM* dermatomyositis, *JDM* juvenile dermatomyositis, *JPM* juvenile polymyositis, *PM* polymyositis, *SD* standard deviation

**Table 2:** Baseline comorbidities and clinical characteristics by cohort

	Cohorts			
	DM (*n* = 4,275)	JDM (*n* = 128)	PM (*n* = 4,559)	JPM (*n* = 47)
Comorbidities^b^, *n* (%)				
BMI, mean (SD)	29.4 (7.6)	22.7 (5.4)	30.0 (9.6)	29.2 (14.2)
Smoking				
Current	1,030 (24.1)	2 (1.6)	1,278 (28.0)	1 (2.1)
Former	263 (6.2)	NA	287 (6.3)	NA
Never	990 (23.2)	38 (29.7)	883 (19.4)	13 (27.7)
Unknown	1,992 (46.6)	88 (68.8)	2,111 (46.3)	33 (70.2)
Asthma	634 (14.8)	15 (11.7)	714 (15.7)	7 (14.9)
ILD	697 (16.3)	10 (7.8)	715 (15.7)	4 (8.5)
ILD requiring oxygen	149 (3.5)	3 (2.3)	191 (4.2)	0 (0.0)
Oxygen supplementation	532 (12.4)	5 (3.9)	824 (18.1)	8 (17.0)
Diabetes	1,116 (26.1)	10 (7.8)	1,541 (33.8)	3 (6.4)
Hyperlipidemia	1,721 (40.3)	6 (4.7)	2,337 (51.3)	3 (6.4)
Hypertension	2,152 (50.3)	9 (7.0)	2,914 (63.9)	6 (12.8)
Heart failure	450 (10.5)	1 (0.8)	799 (17.5)	3 (6.4)
Cardiomyopathy	194 (4.5)	3 (2.3)	297 (6.5)	1 (2.1)
Cardiac dysrhythmia	722 (16.9)	19 (14.8)	1,120 (24.6)	4 (8.5)
Ischemic heart disease	671 (15.7)	1 (0.8)	1,050 (23.0)	0 (0.0)
Pericarditis	42 (1.0)	0 (0.0)	39 (0.9)	0 (0.0)
Myocarditis	16 (0.4)	1 (0.8)	61 (1.3)	1 (2.1)
VTE	336 (7.9)	6 (4.7)	492 (10.8)	4 (8.5)
DVT	174 (4.1)	2 (1.6)	244 (5.4)	2 (4.3)
PE	135 (3.2)	0 (0.0)	199 (4.4)	1 (2.1)
Malignancy, main 6^a^	336 (7.9)	0 (0.0)	341 (7.5)	0 (0.0)
Malignancy, excluding NMSC	779 (18.2)	3 (2.3)	811 (17.8)	1 (2.1)
Polyarthritis	369 (8.6)	13 (10.2)	489 (10.7)	4 (8.5)
CKD	807 (18.9)	6 (4.7)	1,182 (25.9)	10 (21.3)
Liver disease	778 (18.2)	17 (13.3)	984 (21.6)	11 (23.4)
Solid organ transplant	11 (0.3)	0 (0.0)	9 (0.2)	0 (0.0)
Dysphagia	837 (19.6)	20 (15.6)	821 (18.0)	2 (4.3)
Dysphonia	183 (4.3)	1 (0.8)	113 (2.5)	2 (4.3)
Esophageal dysmotility	94 (2.2)	1 (0.8)	91 (2)	0 (0.0)
GERD	1,331 (31.1)	16 (12.5)	1,575 (34.6)	6 (12.8)
Peptic ulcer	149 (3.5)	1 (0.8)	171 (3.8)	1 (2.1)
Aspiration pneumonia	90 (2.1)	1 (0.8)	132 (2.9)	0 (0.0)
MAS	315 (7.4)	6 (4.7)	307 (6.7)	5 (10.6)
Suicidal ideation	13 (0.3)	0 (0.0)	19 (0.4)	1 (2.1)
Clinical Characteristics^b^, n (%)				
Gottron's papules	88 (2.1)	12 (9.4)	4 (0.1)	0 (0.0)
Periungual erythema	147 (3.4)	5 (3.9)	43 (0.9)	1 (2.1)
Raynaud's phenomenon	367 (8.6)	15 (11.7)	361 (7.9)	0 (0.0)
Alopecia areata	26 (0.6)	1 (0.8)	8 (0.2)	0 (0.0)
Cicatricial alopecia	22 (0.5)	0 (0.0)	9 (0.2)	2 (4.3)
Arthritis	206 (4.8)	15 (11.7)	251 (5.5)	5 (10.6)
Antisynthetase syndrome	1 (0.02)^c^	0 (0.0)	2 (0.04)^c^	0 (0.0)
Calcinosis	34 (0.8)	6 (4.7)	7 (0.2)	0 (0.0)
Ulceration	31 (0.7)	2 (1.6)	28 (0.6)	0 (0.0)

^a^ Main 6 malignancies: ovarian, lung, pancreatic, stomach, colorectal, basal or squamous cell

^b^ Assessed during the 12-month period prior to the index date

^c^ The % values have been extended to 2 decimal places to capture the non-zero percentages for antisynthetase syndrome

*BMI* body mass index, *CKD* chronic kidney disease, *DM* dermatomyositis, *DVT* deep vein thrombosis, *GERD* gastroesophageal reflux disease, *ILD* interstitial lung disease, *JDM* juvenile dermatomyositis, *JPM* juvenile polymyositis, *MAS* macrophage activation syndrome, *NA* not applicable, *NMSC* non-melanoma skin cancer, *PE* pulmonary embolism, *PM* polymyositis, *SD* standard deviation, *VTE* venous thromboembolism

**Table 3:** Baseline medications and treatment by cohort

Medications and treatments, *n* (%)	Cohorts			
	DM (*n* = 4,275)	JDM (*n* = 128)	PM (*n* = 4,559)	JPM (*n* = 47)
Systemic steroids	3,005 (70.3)	94 (73.4)	3,115 (68.3)	28 (59.6)
Topical steroids	1,790 (41.9)	46 (35.9)	591 (13.0)	8 (17.0)
Immunosuppressants	2,160 (50.5)	81 (63.3)	1,545 (33.9)	15 (31.9)
Biologics	269 (6.3)	17 (13.3)	270 (5.9)	5 (10.6)
IVIG	967 (22.6)	61 (47.7)	847 (18.6)	13 (27.7)
Combination^a^	1,961 (45.9)	76 (59.4)	1,426 (31.3)	15 (31.9)
Procedure or equipment^b^	1,446 (33.8)	68 (53.1)	2,122 (46.6)	24 (51.1)
Concomitant medication^c^	1,892 (44.3)	11 (8.6)	2,395 (52.5)	4 (8.5)

^a^ Combination: topical or systemic steroids + immunosuppressants or biologic

^b^ Procedure or equipment: physical therapy, heat therapy (microwave or ultrasound), orthotics

^c^ Concomitant medications included the following: pirfenidone, nintedanib, clopidogrel, warfarin, propranolol, diltiazem, isosorbide mononitrate, lisinopril, amlodipine besylate, metoprolol, hydrochlorothiazide, losartan, atorvastatin, simvastatin, pravastatin, fenofibrate, metformin, insulin glargine, glipizide, insulin human, and glimepiride

*DM* dermatomyositis, *IVIG* intravenous immunoglobulin, *JDM* juvenile dermatomyositis, *JPM* juvenile polymyositis, *PM* polymyositis

## Results

### Baseline characteristics

#### Study population and demographics

A total of 9,009 IIM patients were included in the analysis (DM cohort: 4,275; PM cohort: 4,559; JDM cohort: 128; JPM cohort: 47). The mean age at index was 54.5 years for the DM cohort, 57.3 years for the PM cohort, 14.3 years for the JDM cohort, and 15.1 years for the JPM cohort. In all cohorts, the majority of patients were female (DM: 76.1%; PM: 62.5%; JDM: 69.5%; JPM: 55.3%) (Table 1).

#### Comorbidities and clinical characteristics

In both DM and PM cohorts, the three most common baseline comorbidities were hypertension (DM: 50.3%; PM: 63.9%), hyperlipidemia (DM: 40.3%; PM: 51.3%), and gastroesophageal reflux disease (GERD; DM: 31.1%; PM: 34.6%). In the JDM cohort, the most common comorbidities were dysphagia (15.6%), cardiac dysrhythmia (14.8%), and liver disease (13.3%). In the JPM cohort, the most common comorbidities were liver disease (23.4%), chronic kidney disease (CKD; 21.3%), and oxygen supplementation (17.0%) (Table 2).

In both DM and PM cohorts, the three most common baseline clinical characteristics were Raynaud's phenomenon (DM: 8.6%; PM: 7.9%), arthritis (DM: 4.8%; PM: 5.5%), and periungual erythema.

(DM: 3.4%; PM: 0.9%). In the JDM cohort, the most common clinical characteristics were Raynaud's phenomenon and arthritis (11.7% each), and Gottron's papules (9.4%). In the JPM cohort, the most common baseline clinical characteristics were arthritis (10.6%), cicatricial alopecia (4.3%), and periungual erythema (2.1%) (Table 2).

#### Medications and treatments

In both DM and PM cohorts, the most frequently prescribed baseline treatments were systemic steroids (DM: 70.3%; PM: 68.3%). Other frequently prescribed treatments in the DM cohort were immunosuppressants (50.5%), and combination drugs (i.e., topical or systemic steroids plus either immunosuppressants or biologics) (45.9%). Other frequently prescribed treatments in the PM cohort were concomitant medications (52.5%) and procedures (physical or heat therapy) or equipment (orthotics) (46.6%). The most frequently prescribed baseline treatments in the JDM cohort were systemic steroids (73.4%), immunosuppressants (63.3%), and combination drugs (59.4%). In the JPM cohort, these were systemic steroids (59.6%), procedure or equipment (51.1%), and immunosuppressants and combination drugs (31.9% each) (Table 3).

### Outcomes

#### Incidence rates of outcomes of interest

In the DM cohort, the highest IRs (per 100 person years [95% CI]) were observed for GERD.

(10.3 [9.6–11.1]; MC: 6.4 [5.9–7.0]) followed by cardiac dysrhythmia (5.7 [5.2–6.3]; MC: 3.7 [3.3–4.1]), and dysphagia (5.3 [4.9–5.9]; MC: 2.0 [1.8–2.3]). In the PM cohort, the highest IRs were observed for GERD (12.8 [12.0–13.7]; MC: 6.4 [5.9–7.0]), followed by cardiac dysrhythmia (8.0 [7.4–8.6]; MC: 4.3 [4.0–4.8]), and ischemic heart disease (5.7 [5.2–6.2]); MC: 3.1 [2.8–3.4]) (Table 4).

**Table 4:** Incidence rates of selected outcomes: DM and PM cohorts versus matched controls

	IR (95% CI)		IR (95% CI)	
Outcomes	DM cohort	Matched controls	PM cohort	Matched controls
ILD	2.8 (2.4–3.1)	0.5 (0.4–0.6)	2.4 (2.1–2.7)	0.5 (0.4–0.6)
ILD requiring oxygen	0.8 (0.6–1.0)	0.1 (0.0–0.2)	0.9 (0.7–1.1)	0.1 (0.1–0.2)
Heart failure	2.8 (2.5–3.2)	1.6 (1.3–1.8)	4.2 (3.9–4.7)	2.0 (1.8–2.3)
Cardiomyopathy	1.2 (1.0–1.5)	0.7 (0.6–0.9)	2.0 (1.8–2.3)	0.8 (0.6–1.0)
Cardiac dysrhythmia	5.7 (5.2–6.3)	3.7 (3.3–4.1)	8.0 (7.4–8.6)	4.3 (4.0–4.8)
Ischemic heart disease	4.0 (3.6–4.4)	2.3 (2.0–2.6)	5.7 (5.2–6.2)	3.1 (2.8–3.4)
Dysphagia	5.3 (4.9–5.9)	2.0 (1.8–2.3)	5.3 (4.8–5.8)	1.9 (1.6–2.1)
Esophageal dysmotility	0.7 (0.5–0.8)	0.1 (0.1–0.2)	0.7 (0.5–0.8)	0.1 (0.1–0.2)
GERD	10.3 (9.6–11.1)	6.4 (5.9–7.0)	12.8 (12.0–13.7)	6.4 (5.9–7.0)
Peptic ulcer	1.4 (1.2–1.7)	0.6 (0.4–0.7)	1.3 (1.1–1.5)	0.5 (0.4–0.7)
Malignancy, excluding NMSC	2.5 (2.2–2.8)	1.7 (1.4–1.9)	2.4 (2.1–2.7)	1.8 (1.6–2.1)
Malignancy, main 6^a^	1.7 (1.5–2.0)	1.1 (0.9–1.3)	1.8 (1.6–2.1)	1.4 (1.2–1.6)
All-cause death	2.2 (1.9–2.5)	0.8 (0.6–0.9)	2.6 (2.4–3.0)	1.0 (0.9–1.2)

IRs calculated as IR (95% CI) per 100 person years; mean follow-up period was generally 2 to 3 years

^a^ Main 6 malignancies: ovarian, lung, pancreatic, stomach, colorectal, basal or squamous cell

*CI* confidence interval, *DM* dermatomyositis, *GERD* gastroesophageal reflux disease, *ILD* interstitial lung disease, *IR* incidence rates, *NMSC* non-melanoma skin cancer, *PM* polymyositis

In the JDM cohort, dysphagia had the highest IR (4.3 [2.3–8.1]; MC: 0.0 [0.0–1.5]) followed by GERD (3.9 [2.1–7.5]; MC: 0.8 [0.3–3.1]), and cardiac dysrhythmia (3.6 [1.9–7.2]; MC: 0.4 [0.1–2.2]). In the JPM cohort, cardiac dysrhythmia had the highest IR (3.5 [1.3–10.1]; MC: 2.4 [0.8–8.8]) followed by cardiomyopathy (2.2 [0.7–8.0]; MC: 0.0 [0.0–4.3]), and dysphagia (2.2 [0.7–7.9]; MC: 0.0 [0.0–4.3]) (Table 5).

**Table 5:** Incidence rates of selected outcomes: JDM and JPM cohorts versus matched controls

	IR (95% CI)		IR (95% CI)	
Outcomes	JDM cohort	Matched controls	JPM cohort	Matched controls
ILD	1.2 (0.4–3.5)	0.0 (0.0–1.5)	1.2 (0.3–6.6)	0.0 (0.0–4.3)
ILD requiring oxygen	0.4 (0.1–2.1)	0.0 (0.0–1.5)	0.0 (0.0–3.9)	0.0 (0.0–4.3)
Heart failure	0.4 (0.1–2.1)	0.0 (0.0–1.5)	0.0 (0.0–4.1)	0.0 (0.0–4.3)
Cardiomyopathy	0.8 (0.2–2.8)	0.0 (0.0–1.5)	2.2 (0.7–8.0)	0.0 (0.0–4.3)
Cardiac dysrhythmia	3.6 (1.9–7.2)	0.4 (0.1–2.2)	3.5 (1.3–10.1)	2.4 (0.8–8.8)
Ischemic heart disease	0.0 (0.0–1.4)	0.0 (0.0–1.5)	0.0 (0.0–3.9)	0.0 (0.0–4.3)
Dysphagia	4.3 (2.3–8.1)	0.0 (0.0–1.5)	2.2 (0.7–7.9)	0.0 (0.0–4.3)
Esophageal dysmotility	0.8 (0.2–2.8)	0.0 (0.0–1.5)	0.0 (0.0–3.9)	0.0 (0.0–4.3)
GERD	3.9 (2.1–7.5)	0.8 (0.3–3.1)	1.2 (0.3–6.9)	0.0 (0.0–4.3)
Peptic ulcer	0.4 (0.1–2.1)	0.0 (0.0–1.5)	0.0 (0.0–3.9)	0.0 (0.0–4.3)
Malignancy, excluding NMSC	0.0 (0.0–1.4)	0.0 (0.0–1.5)	0.0 (0.0–4.0)	0.0 (0.0–4.4)
Malignancy, main 6^a^	0.0 (0.0–1.4)	0.0 (0.0–1.5)	0.0 (0.0–3.9)	0.0 (0.0–4.3)
All-cause death	0.4 (0.1–2.1)	0.0 (0.0–1.5)	0.0 (0.0–3.9)	0.0 (0.0–4.3)

IRs calculated as IR (95% CI) per 100 person years; mean follow-up period was generally 2 to 3 years

^a^ Main 6 malignancies: ovarian, lung, pancreatic, stomach, colorectal, basal or squamous cell

*CI* confidence interval, *GERD* gastroesophageal reflux disease, *ILD* interstitial lung disease, *IR* incidence rates, *JDM* juvenile dermatomyositis, *JPM* juvenile polymyositis, *NMSC* non-melanoma skin cancer

#### Progression times to outcomes of interest

In the DM and PM cohorts, the outcomes with the shortest mean time to event were GERD (1.3 years) and ILD (1.2 years), respectively. Outcomes with the longest mean time to event were heart failure in the DM cohort (1.6 years) and all-cause death in the PM cohort (1.7 years). Outcomes with the shortest and longest mean time to event were cardiomyopathy (0.4 years) and peptic ulcer (1.6 years), respectively, in the JDM cohort and ILD (0.2 years) and cardiomyopathy (2.8 years), respectively, in the JPM cohort (Online Resource 1).

#### Predictors of incident interstitial lung disease, malignancy, and all-cause death

The parameters of the random forest models predicting ILD, malignancies, and all-cause death outcomes are shown in Online Resource 2. The strongest predictors for ILD were immunosuppressants and older age in the DM cohort and Raynaud’s phenomenon and topical steroids in the PM cohort. The strongest predictors for malignancy were older age and hyperlipidemia in the DM cohort and older age and heart failure in the PM cohort. The strongest predictors of all-cause death were older age and heart failure in both DM and PM cohorts (Online Resource 3).

## Discussion

DM and PM are severe autoimmune diseases, whose rarity has led to gaps in understanding patient demographics, comorbidities, clinical characteristics, treatment exposures, and disease progression patterns, including extra-muscular manifestations and malignancy risks. This retrospective natural history study included 9,009 patients with diagnoses of DM (*n* = 4,275), PM (*n* = 4,559), JDM (*n* = 128), and JPM (*n* = 47) and an equal number of MCs in each cohort. To our knowledge, this is the largest sample of DM and PM patients assembled in a single study to date. All sub-types were found to be more prevalent in females than in males, consistent with the published literature [3, 4, 6–11].

### Baseline characteristics

#### Comorbidities

The most common comorbidities in the DM and PM cohorts in our study were hypertension, hyperlipidemia, and GERD. The most common comorbidities in the JDM cohort in our study were dysphagia, cardiac dysrhythmia, and liver disease, and in the JPM cohort were liver disease, chronic kidney disease, and oxygen supplementation. Similar findings were reported in a recent US study of prevalent DM patients in which the most common comorbidities were hypertension, other autoimmune diseases, GERD, and dyslipidemia [7]. In a retrospective study in South Korea, the most common comorbidities in a composite group of prevalent DM, PM, and JDM patients were chronic pulmonary disease, hypertension, and liver disease. Cardiac arrhythmia was far less prevalent than in our patient cohorts, and hyperlipidemia, dysphagia, and GERD were not reported [5]. In a US study of prevalent JDM and JPM patients, prevalence of dysphagia was notably higher in both patient groups compared with our study [26].

#### Clinical characteristics

The most common clinical characteristics in the DM and PM cohorts in our study were Raynaud's phenomenon, arthritis, and periungual erythema. In a retrospective study in China of prevalent DM and PM patients, the prevalence of Raynaud’s phenomenon was lower compared with our study [27]. In the JDM cohort in our study, the most common clinical characteristics were Raynaud's phenomenon, arthritis, and Gottron's papules. By comparison, in a US study of prevalent JDM patients, Gottron’s papules and arthritis were far more prevalent than in our study, but prevalence of Raynaud’s phenomenon was comparable [26]. In the JPM cohort in our study, the most common clinical characteristic was arthritis, although it was far more prevalent in the US study of prevalent JPM patients [26]. The variation in clinical characteristics across studies may be partially attributed to variability in geographic region, patient samples (incident versus prevalent), duration of follow-up, data sources (e.g., administrative databases may be less likely to capture disease symptoms than more bespoke or single-centre data sources), and diagnostic criteria across different study time periods.

#### Medications and treatment

Systemic steroids were the most frequently prescribed medications and treatment in our study for all cohorts. Similar findings were observed in a multicenter cohort study of prevalent adult and juvenile myositis patients in Spain [28], a retrospective study of prevalent adult and juvenile myositis patients in England [29], and a multicenter study of JDM patients in Europe and Latin America [30].

### Outcomes

For all outcomes, the patients with DM and PM had substantially higher IRs than their MCs. In the JDM and JPM cohorts, outcomes were less frequent compared with the adult DM and PM cohorts, and for many outcomes, none were observed during follow-up. When outcomes did occur, IRs were consistently higher in the JDM and JPM cohorts compared with their MCs. In DM and PM cohorts, the highest IRs were observed for GERD and cardiac dysrhythmia. In the JDM cohort, dysphagia and GERD had the highest IRs and in the JPM cohort, cardiac dysrhythmia and cardiomyopathy had the highest IRs.

Few studies with comparable designs and outcomes are available in which meaningful comparisons with this study’s IRs can be made. In a retrospective cohort study in Japan with prevalent adult and juvenile DM and PM patients, both groups had markedly higher ILD IRs than in our study [8]. In contrast, a retrospective study in Taiwan with incident adult and juvenile DM and PM patients reported markedly lower ILD IRs than in our study [31]. However, in both the current study and the study in Taiwan, the DM and PM patients had considerably higher ILD rates than the MCs. In a retrospective study in China, DM patients had a higher death rate and were more likely to have malignancy and ILD compared with the PM patients [27]. By comparison, the DM and PM patient groups had similar rates of all three outcomes in our study. The high IRs of cardiovascular conditions observed in the DM and PM cohorts in our study, relative to MCs, have also been noted elsewhere [32, 33]. In a population-based study in Denmark, patients with DM or PM experienced more frequent left ventricular diastolic dysfunction (LVDD) compared with the healthy controls [34]. Similar findings were reported in a study in China including DM patients without any evident cardiovascular disease [35]. In a nationwide inpatient study in the US, among patients with atherosclerotic cardiovascular disease, those with DM had almost double in-hospital mortality compared with their MCs without DM [36]. In a study in Canada, incident cardiovascular events were found to be higher in patients with DM and PM compared with the rates in the general Canadian population [37]. In a study in Norway including patients with JDM, diastolic dysfunction was reported in 22% of patients with JDM versus 0% of controls at a median of 16.8 years after diagnosis [38].

#### Predictors of incident interstitial lung disease, malignancy, and all-cause death

Our study identified advanced age as the strongest predictor of malignancy and all-cause death in both DM and PM cohorts and as one of the top five predictors of ILD in both cohorts. Apart from age, other predictors of ILD and malignancy varied across these cohorts. Heart conditions and malignancies were identified as other predictors of all-cause death. Similar findings were reported in two studies in Spain in which major predictors of all-cause death were cardiovascular events and cancer in both DM and PM patients [28, 39]. However, in a retrospective study from England, pulmonary involvement was identified as the only statistically significant independent predictor of all-cause death [29].

### Limitations

Our study has several limitations. First, some misclassification of the DM and PM patient groups and their baseline characteristics and outcomes is expected. Regarding DM and PM classifications, our algorithm has not been validated and confirming each diagnosis against clinical notes and labs was not within the scope and resources of this study. Further, the DM and PM disease categories often encompass more specific subtypes or recently recognized IIM diseases for which standardized biomarkers and ICD codes did not exist during the study period; thus, the results pertain to the broader DM and PM disease categories and not necessarily to subtypes [23]. Additionally, symptoms may be misclassified through coding errors and under-coding (pathognomonic symptoms such as Gottron’s papules in dermatomyositis patients may be particularly susceptible to under-coding). Second, small sample sizes in the juvenile JDM and JPM cohorts resulted in infrequent outcome events in these groups, resulting in imprecise IR estimates. Third, our JDM and JPM results are limited to juvenile cases who were first diagnosed at age 12 or older and may not generalize to those diagnosed at younger ages. Fourth, limited follow-up time generally restricted our observations to the first two to three years following the initial diagnosis with DM, PM, JDM, and JPM. Fifth, we lacked race, ethnicity, and smoking status data on a substantial proportion of study patients – ranging from 19.5% for race for DM patients to 70.2% for smoking status for JPM patients. We did not impute values, and this degree of missing data precludes drawing conclusions about race, ethnicity, and smoking history distributions in our sample and in the broader population of DM, PM, JDM, and JPM patients in the US. In addition, missing data undermined how robustly these variables could predict outcomes in the random forest analyses.

## Conclusion

To our knowledge, this is the largest study yet to characterize DM and PM patients across a range of demographic and disease characteristics. The results of this real-world study often align with the findings from other smaller studies of IIM patients. Specifically, the ages and sex distributions of the patients, their comorbidities, treatment patterns, and outcomes were often consistent with these previous studies. Additionally, this study addresses an essential gap in existing knowledge of the epidemiology of incident DM and PM in the US, namely, IRs of multiple extra-muscular disease manifestations and malignancy events. Our findings contribute to a growing literature on IIM patients’ clinical characteristics and disease progression patterns that may provide valuable insights to clinicians treating IIM patients, leading to more individualized treatment strategies that better anticipate disease progression. For example, better understanding the risks of extra-muscular disease progression and the predictors of ILD, malignancy, and all-cause death in IIM patients may provide clinicians a basis for more individualized patient surveillance and a better rationale for specialized testing, including high-resolution CT to detect ILD and MRI to enhance early detection of malignancies. We conclude that DM and PM patients in our sample, compared with their MCs, had appreciably higher IRs of extra-muscular IIM disease manifestations, pointing to these patients’ unmet treatment needs. Given this study’s large sample of adult patients from across the 50 states, the results reported here may fairly generalize to adult DM and PM patients in the US.

## Supplementary Information

Below is the link to the electronic supplementary material.

Supplementary file1 (DOCX 86.3 KB)

## Data Availability

Due to data license agreement restrictions, the data that supported this study are not publicly available.
