# Correction: A retrospective natural history study in adult and juvenile patients with incident dermatomyositis and polymyositis using real world data

**DOI:** 10.1007/s10067-025-07800-6

**Published:** 2025-11-20

**Authors:** David M. Barnes, Daniela Graham, Cecilia E. Borlenghi, Thomas Edwards, Stephen E. Schachterle, Helen Sile

**Affiliations:** 1 Pfizer Inc, MS 66HB-13N251/261, 66 Hudson Boulevard East, New York, NY 10001 USA; 2 Pfizer Inc, Groton, CT USA; 3 Pfizer Inc, Buenos Aires, Argentina; 4 Panalgo Inc, Boston, MA USA

**Correction: Clinical Rheumatology (2025) 44:4237–4247**

10.1007/s10067-025-07614-6

The copyright holder for this article was incorrectly given as '© The Author(s), under exclusive licence to International League of Associations for Rheumatology (ILAR) 2025' but should have been '© Pfizer 2025'.

The original article has been corrected.

