# Supplementary material for: A retrospective natural history study in adult and juvenile patients with incident dermatomyositis and polymyositis using real world data
Source: Clin Rheumatol. 2025 Aug 28;44(10):4237–47. doi: 10.1007/s10067-025-07614-6 (PMC12634748; doi:10.1007/s10067-025-07614-6)
Supplement: Supplementary file 1 — Supplementary file1 (DOCX 86.3 KB) [file 10067_2025_7614_MOESM1_ESM.docx]

# **A Retrospective Natural History Study in Adult and Juvenile Patients with Incident Dermatomyositis and Polymyositis Using Real World Data**

**Authors:** David M. Barnes^1*^, Daniela Graham^2^, Cecilia E. Borlenghi^3^, Thomas Edwards^4^, Stephen E. Schachterle^1^, Helen Sile^1^

**Affiliations:** ^1^ Pfizer Inc., New York, NY, USA; ^2^ Pfizer Inc., Groton, CT, USA; ^3^ Pfizer Inc., Buenos Aires, Argentina; ^4^ Panalgo Inc., Boston, MA, USA

**Corresponding Author:**

* David M. Barnes

MS 66HB-13N251/261

66 Hudson Boulevard East

New York New York 10001, US

**Phone Number**: 646-306-8024

**E-mail:** [david.barnes@pfizer.com](mailto:david.barnes@pfizer.com)

**Target Journal:** Clinical Rheumatology

**Supplementary Tables**

## **Online Resource 1. Progression Times to Outcomes by Cohort**

|  | **Cohorts** | | | |
| --- | --- | --- | --- | --- |
| **Outcomes, mean years to event^a^** | **DM (n=4,275)** | **JDM  (n=128)** | **PM (n=4,559)** | **JPM**  **(n=47)** |
| ILD | 1.3 | 1.2 | 1.2 | 0.2 |
| ILD requiring oxygen | 1.3 | 0.6 | 1.4 | NA |
| Heart failure | 1.6 | 0.6 | 1.5 | NA |
| Cardiomyopathy | 1.5 | 0.4 | 1.5 | 2.8 |
| Cardiac dysrhythmia | 1.5 | 1.0 | 1.5 | 1.8 |
| Ischemic heart disease | 1.5 | NA | 1.5 | NA |
| Dysphagia | 1.4 | 1.1 | 1.4 | 0.9 |
| Esophageal dysmotility | 1.4 | 0.5 | 1.5 | NA |
| GERD | 1.3 | 1.2 | 1.3 | 1.0 |
| Peptic ulcer | 1.3 | 1.6 | 1.6 | NA |
| Malignancy, excluding NMSC | 1.4 | NA | 1.6 | NA |
| Malignancy, main 6^b^ | 1.5 | NA | 1.5 | NA |
| All-cause death | 1.5 | 0.8 | 1.7 | NA |

^a^ Mean follow-up period was generally 2 to 3 years

^b^ Main 6 malignancies: ovarian, lung, pancreatic, stomach, colorectal, basal or squamous cell

DM, dermatomyositis; GERD, gastroesophageal reflux disease; ILD, interstitial lung disease; JDM, juvenile dermatomyositis; JPM, juvenile polymyositis; NA, not applicable (these events did not occur in this sample during the study follow-up time); NMSC, non-melanoma skin cancer; PM, polymyositis

## **Online Resource 2. Parameters used in random forest models for ILD, Malignancy, and All-Cause Death by cohort (DM and PM)**

| **Parameters** | **Value** | | | | | |
| --- | --- | --- | --- | --- | --- | --- |
|  | **ILD** | | **Malignancy** | | **All-cause death** | |
|  | **DM** | **PM** | **DM** | **PM** | **DM** | **PM** |
| Area under curve (AUC) | 65.42% | 60.62% | 61.02% | 63.89% | 82.11% | 75.21% |
| 95% CIs around AUC | 59.25%-71.58% | 54.36%-66.88% | 54.73%-67.31% | 57.42%-70.36% | 77.62%-86.60% | 70.60%-79.81% |
| Recall | 79.01% | 41.98% | 47.22% | 58.57% | 84.09% | 67.89% |
| Precision | 10.42% | 9.14% | 8.88% | 8.93% | 15.42% | 15.10% |
| Negative predictive value | 96.30% | 93.98% | 94.29% | 95.64% | 98.25% | 96.01% |
| Specificity | 44.56% | 68.47% | 64.24% | 60.34% | 66.00% | 66.96% |

AUC, area under curve; DM, dermatomyositis; ILD, interstitial lung disease; PM, polymyositis

## **Online Resource 3. Strongest Predictors of ILD, Malignancy, and All-Cause Death by Cohort Using Mean Decrease in Impurity Metric**

| **Outcomes** | **Strongest Predictors (MDI, %)** | |
| --- | --- | --- |
|  | **DM Cohort (n=4,275)** | **PM Cohort (n=4,559)** |
| **ILD** | Immunosuppressants (9.6) | Raynaud’s phenomenon (19.0) |
|  | Age (8.7) | Topical steroids (11.4) |
|  | Region: South^a^ (6.2) | Malignancy, main 6 (10.0) |
|  | Esophageal dysmotility (4.8) | Age (9.7) |
|  | Oxygen supplementation (3.8) | Pericarditis (7.3) |
| **Malignancy, excluding NMSC** | Age (40.8) | Age (12.3) |
|  | Hyperlipidemia (8.1) | Heart failure (11.3) |
|  | Dysphagia (7.1) | Current smoker (9.6) |
|  | Hypertension (5.0) | Female^a^ (7.5) |
|  | Current smoker (4.4) | MAS^a^ (6.5) |
| **All-cause death** | Age (15.9) | Age (24.3) |
|  | Heart failure (8.4) | Heart failure (12.8) |
|  | Cardiac dysrhythmia (7.6) | Ischemic heart disease (8.2) |
|  | Oxygen supplementation (7.0) | Aspiration pneumonia (5.3) |
|  | Malignancy, main 6^b^ (5.4) | Malignancy, excluding NMSC (4.8) |

^a^ Negative association with the outcome, i.e., the factor was associated with a lower risk of the outcome relative to not having the factor or relative to the reference group

^b^ Main 6 malignancies: ovarian, lung, pancreatic, stomach, colorectal, basal or skin squamous cell

DM, dermatomyositis; ILD, interstitial lung disease; MAS, macrophage activation syndrome; MDI, Mean Decrease in Impurity Metric; NMSC, non-melanoma skin cancer; PM, polymyositis
